# Werner Irnich: Pioneer in the Development of the Dual‐Chamber Pacemaker—An Obituary

**DOI:** 10.1111/pace.70269

**Published:** 2026-05-02

**Authors:** Bernd Lemke

**Affiliations:** ^1^ Department of Cardiology, Electrophysiology and Angiology Märkische Kliniken Lüdenscheid Germany; ^2^ Medical Faculty Ruhr University Bochum Bochum Germany

**Keywords:** chronaxie rheobase, interference immunity, optimal pacemaker, pacemaker register, Werner Irnich

## Abstract

Fifty years ago, Werner Irnich presented the concept of an optimal pacemaker capable of responding appropriately to various cardiac arrhythmias and perceptual disturbances, and intended to be used in 85% of patients. With this concept, Irnich was far ahead of his time. His proposed circuitry for AV block and atrial fibrillation, as well as his suggestions for antitachycardia pacing and interference detection, were visionary. In the field of rate‐adaptive pacing, he introduced AV‐time control, the first closed‐loop system. Werner Irnich represents the close connection between engineers and physicians in the field of cardiac electrotherapy. His theoretical work on the chronaxie rheobase and the electrode surface, confirmed by experimental data, still forms the basis of modern electrical stimulation today. The most extensive data on the interference immunity of electronic implants comes from his laboratory. In addition to his membership in numerous scientific societies, Werner Irnich served as Senior Editor of the international journal Pacing and Clinical Electrophysiology (PACE) from 1978 to 2013. He passed away on December 2, 2023, at the age of 89, leaving behind his wife Hanni, five children, and twelve grandchildren. We will always remember him with gratitude and deep appreciation for his contributions to cardiac electrostimulation.

**Abbreviations**
AV: atrioventricular
DDD: dual‐chamber pacing and sensing pacemaker, demand and triggered function
ECG: electrocardiogram
RWTH: Rheinisch‐Westfälische Technische Hochschule
SA: sinoatrial

## 1 The Optimal Pacemaker

Fifty years ago, Werner Irnich introduced the concept of the “Optimal Pacemaker,” a device designed to respond appropriately to a wide range of cardiac arrhythmias and sensing issues: AV block with sinus rhythm, AV block with atrial fibrillation or flutter, AV block with sinus bradycardia or SA block, isolated sinus bradycardia or SA block, the occurrence of ventricular extrasystoles, atrial or ventricular tachycardias, and sensing disturbances at both the atrial and ventricular level. He presented this concept in May 1975 in *Biomedizinische Technik, Volume 20 (Supplement)* [1], based on a lecture he had given at the annual meeting of the German Society for Biomedical Engineering in Stuttgart.

*“The fundamental principle of this pacemaker consists of two almost identical channels, each connected either to the atrium or to the ventricle. Each channel is made up of: 1. amplifier, 2. filter and interference detection, 3. refractory circuit, 4. pulse shaper, and 5. pulse generator.”* (Figure 1).

**FIGURE 1:**
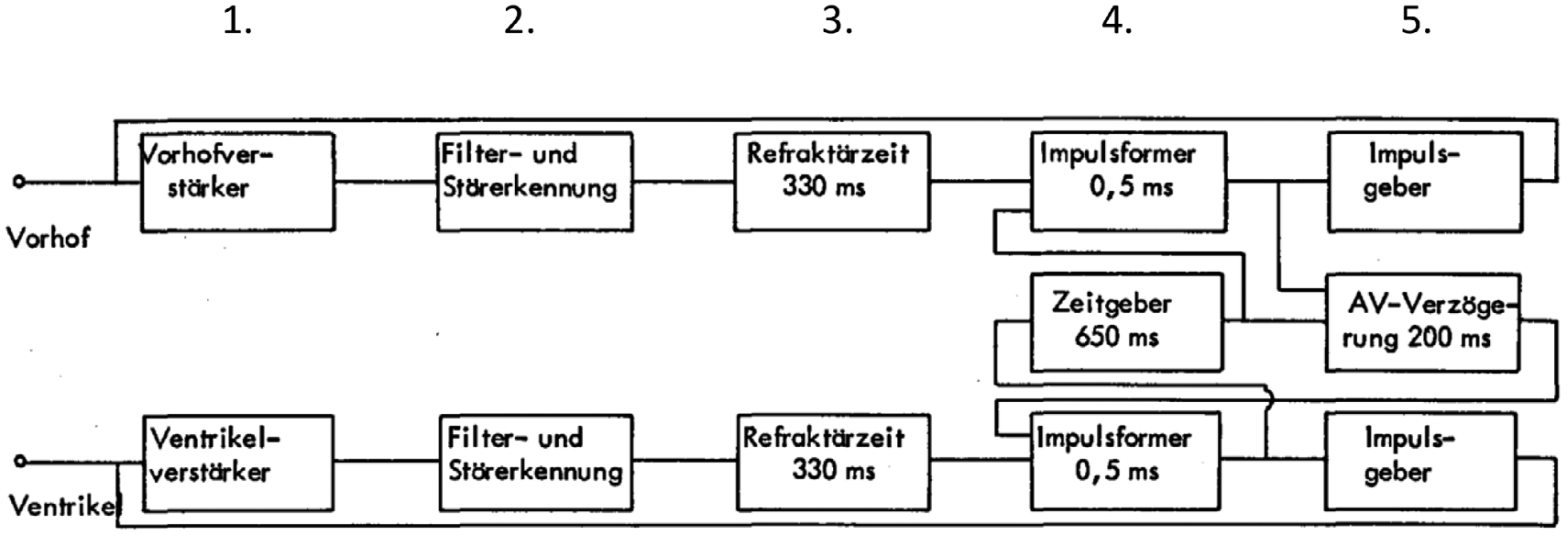
Block diagram of a universal pacemaker. Vorhof= atrium; Ventrikel= ventricle; Zeitgeber= timer; AV‐Verzögerungszeit= AV‐delay. Irnich W, de Bakker JMT. Konzept eines Optimal‐Schrittmachers. Biomedizinische Technik 1975; 20(Ergänzungsband):89–90.

The dual‐chamber pacemaker was ventricle‐driven with a lower limit rate of 70 bpm, determined by a timing circuit of 650 ms and an AV delay of 200 ms. In cases of AV block with sinus rhythm, the atrial refractory period of 330 ms limited the maximum tracking rate to 180 bpm. A particularly forward‐looking feature was the proposed circuit for AV block with atrial fibrillation: if the PP interval was less than 330 ms, the atrial refractory period was extended to 600 ms, thereby limiting the ventricular pacing rate to 100 bpm.

For ventricular extrasystoles, simultaneous atrial and ventricular stimulation was intended to prevent the initiation of reentry tachycardias. Atrial and ventricular tachycardias were to be controlled by coupled stimulation, effectively halving the heart rate (see below). Simultaneous sensing of signals at both the atrial and ventricular inputs was interpreted as noise, in order to avoid inappropriate inhibition of pacing output.

In AV block with sinus rhythm—which Irnich estimated to occur in 59.5% of cases—the result was atrial triggered ventricular pacing, with impulses synchronized to the detected atrial activity. This triggered atrial pacing may have been chosen for patent‐related reasons (see Berkovits’ patent below), but it had the additional benefit of preventing asynchronous ventricular pacing when atrial sensing was inadequate—thus reducing the risk of reentry tachycardias.

In AV block with sinus node disease (12.3%), sequential atrial and ventricular pacing was applied. In isolated sinus node disease (13.8%), pure atrial pacing was used, with the rate increasing according to spontaneous AV conduction. A simulation of the different pacing modes is shown in Figure 2. According to current nomenclature, the pacemaker mode would be described as DDT(A)D(V). However, the official pacemaker code only included the DDD mode starting in 1981 [3].

**FIGURE 2:**
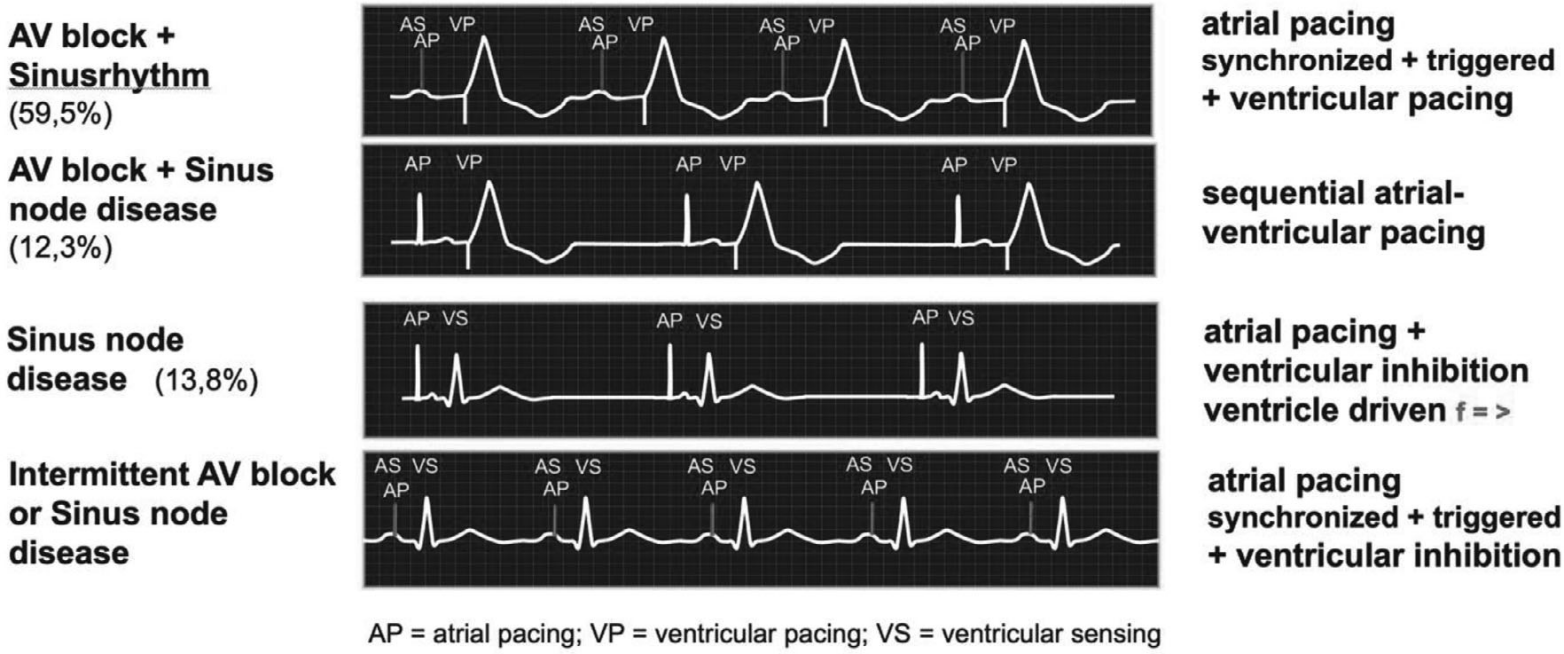
Simulation of different pacing modes in the surface ECG. Triggered atrial pacing is highlighted in red. *f* = > increase in pacing frequency depending on AV conduction. Lemke, B. Werner Irnich and the development of the dual‐chamber pacemaker. Herzschr Elektrophys 2025; 36:265–274.

The optimal pacemaker was intended for use in 85% of patients. *“Except in cases of bradycardia associated with atrial fibrillation or flutter, and in rare instances of intermittent AV block (about 15% of all cases), where the standard demand pacemaker is entirely sufficient, a pacemaker version would be desirable that could be applied to all other diagnoses.”* As the simulation in Figure 2 demonstrates, the optimal pacemaker would also have been suitable for intermittent AV block or sinus node disease.

Werner Irnich never patented his concept. The reasons for this decision remain unclear. Two years later, Hermann Funke, a cardiac surgeon from Bonn, Germany, patented the “universal pacemaker” together with Medtronic [4]. In his application, Irnich's work was not mentioned, and it had never been published outside the journal *Biomedizinische Technik*. Instead, Funke's patent referred to Berkovits as the inventor of the dual‐chamber demand pacemaker—although Berkovits’ design did not allow for atrial triggered ventricular pacing [5]. This capability, however, was present in both Irnich's and Funke's concepts.

In contrast to Irnich's optimal dual‐chamber pacemaker—ventricle‐driven and featuring triggered atrial pacing [DDT(A)D(V)]—Funke's universal pacemaker was atrium‐driven, with a demand circuit in both atrium and ventricle (DDD) [6]. While Irnich's block diagram still relied on an analog circuit, Funke's patent already proposed a digital design.

Irnich was far ahead of his time with the concept of the optimal pacemaker—particularly with regard to his proposals for antitachycardia pacing and noise detection.

## 2 The Tachycardia Stimulator

The method of coupled antitachycardia stimulation originated with the treatment of a patient named Francki, who had sustained thoracic trauma in a traffic accident and subsequently developed paroxysmal ventricular tachycardias with rates of 160–180 bpm. Despite exhaustive treatment attempts with all available medications of the time, therapy was unsuccessful.

In 1967, he was referred to Prof. Sven Effert at RWTH Aachen. After further unsuccessful pharmacological trials, Effert attempted the method of coupled stimulation. When this approach proved to be effective, a temporary stimulator was ordered from the company Elema and delivered in 1969. Connected to an electrode in the right ventricle and a counter‐electrode positioned at the cardiac apex, the device failed to meet expectations.

Following extensive measurements, a new stimulator concept was developed, incorporating a differentiating amplifier characteristic and delivering an ECG‐triggered, delayed stimulation impulse. The modified device, designed by Irnich and named the “Francki Stimulator” after the patient, was described by Irnich himself as his “journeyman piece” (Figure 3). It was implanted on October 26, 1970, and functioned satisfactorily until 1977. From 1976 onward, the patient no longer required the device [7].

**FIGURE 3:**
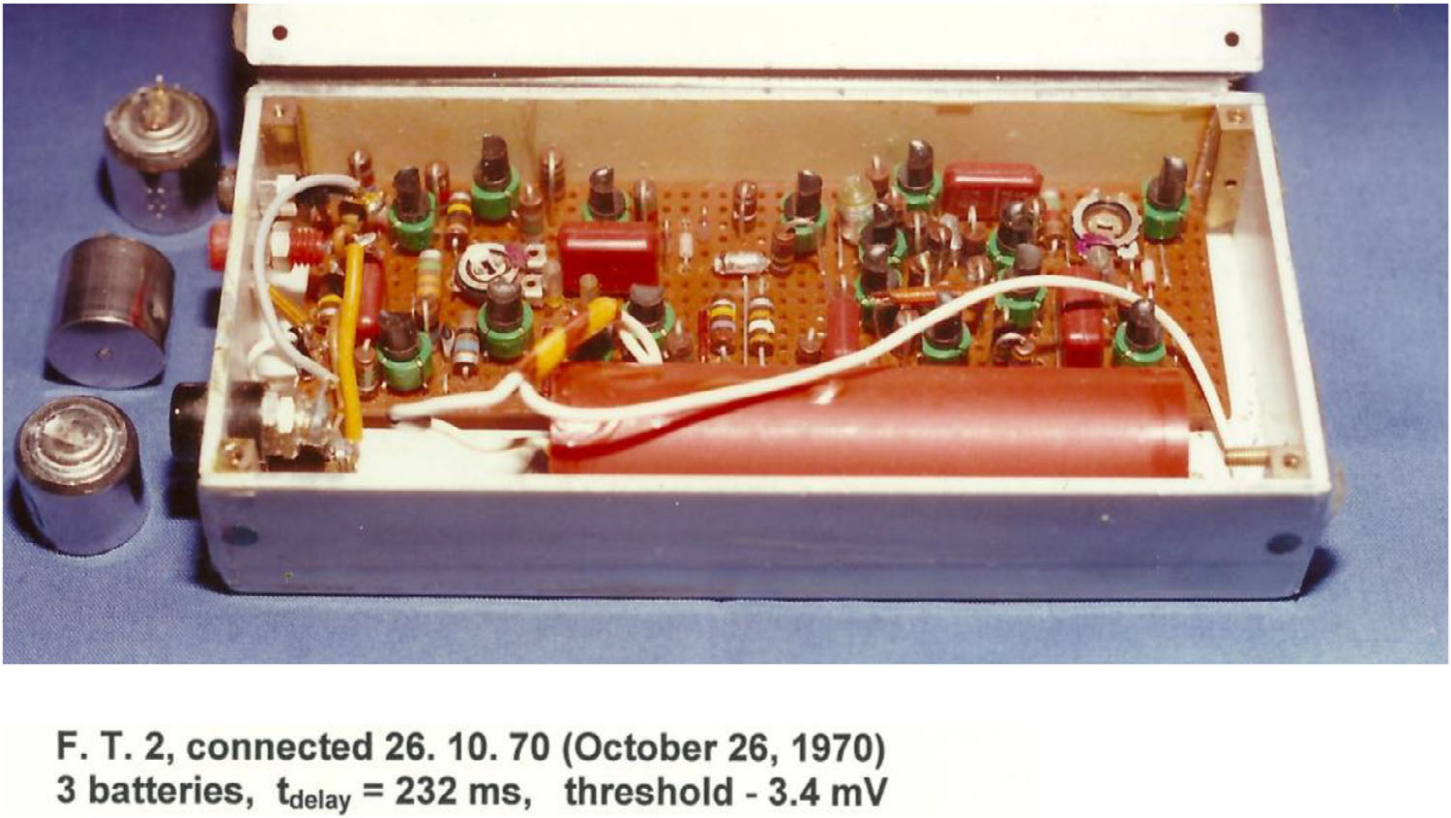
The Francki Stimulator—original description. Irnich W. 50th anniversary celebration of the Medical Faculty RWTH Aachen 2016 “When medical technology came to Aachen”. [Colour figure can be viewed at wileyonlinelibrary.com]

The principle of coupled stimulation is that, when a ventricular tachycardia occurs and a pathological electrogram is detected (Figure 4), the myocardium is stimulated precisely at the point when the earliest depolarization is possible right after the absolute refractory period—without producing a hemodynamic effect. At the same time, every second impulse originating from the arrhythmogenic focus is blocked by tissue that has already depolarized. This results in a pulse rate exactly equal to half the tachycardia frequency (Figure 5).

**FIGURE 4:**
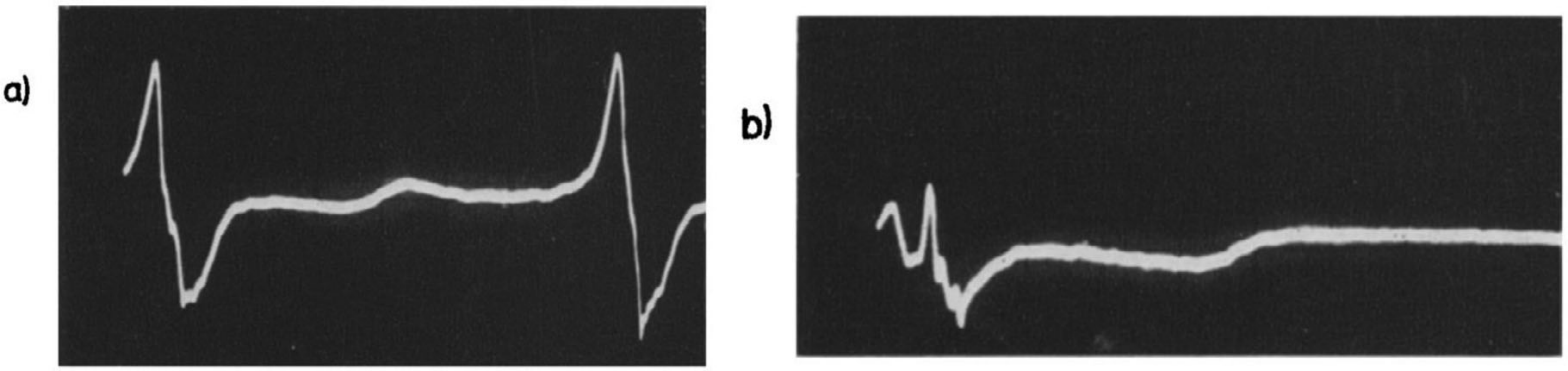
Differences in the intracardiac ECG: (a) ventricular tachycardia, (b) sinus rhythm. Merx M, Irnich W, Effert S, Bleifeld W. Technical note. A triggered generator for the electrical treatment of tachycardias. Med & biol Engng 1972; 10:297–300.

**FIGURE 5:**
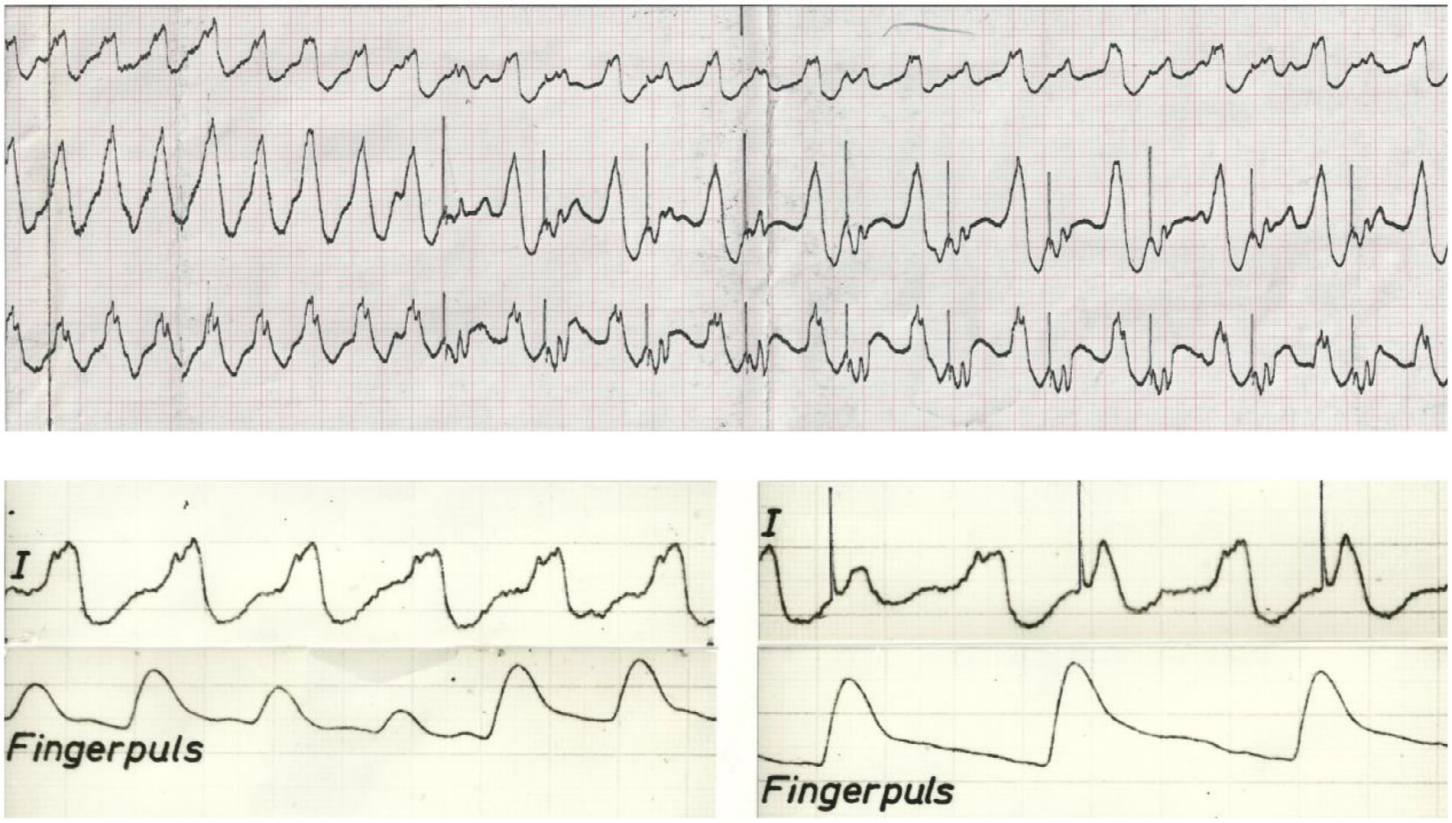
Principle of coupled antitachycardia stimulation—halving of the tachycardia rate. Top: Electrocardiogram without and with electrical stimulation (leads I, II, III; March 31, 1969). Bottom: Hemodynamic effect without and with stimulation (Fingerpuls = finger pulse). Irnich W. 50th anniversary celebration of the Medical Faculty RWTH Aachen 2016 “When medical technology came to Aachen”. [Colour figure can be viewed at wileyonlinelibrary.com]

The results of this stimulation method were presented by Merx, Irnich, Effert, and Bleifeld in August 1971 at the International Conference in Melbourne. The improvement in cardiac insufficiency observed in four patients treated with this approach was remarkable. In two patients, ventricular tachycardia could be terminated with coupling intervals of 220 and 180 ms, respectively. At the same time, triggered stimulation delivered into the ventricular extrasystole successfully prevented the recurrence of tachycardia [8] (Figure 6).

**FIGURE 6:**
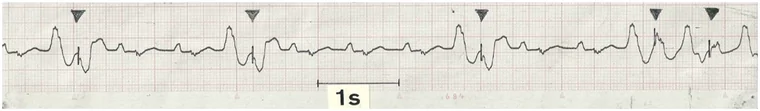
Prevention of ventricular tachycardia onset (first three stimuli). Prolongation of the coupling interval with the fourth stimulus leads to recurrence of tachycardia. Merx M, Irnich W, Effert S, Bleifeld W. Technical note. A triggered generator for the electrical treatment of tachycardias. Med & biol Engng 1972; 10:297–300. [Colour figure can be viewed at wileyonlinelibrary.com]

The authors also pointed out a potential risk of the method: stimulation during the “vulnerable phase” could induce ventricular fibrillation, particularly in the presence of ischemic heart disease. For this reason, the pulse duration was limited to 1 ms, and the pulse amplitude was not set higher than 4 V.

## 3 The Rate‐Adaptive Pacemaker

The need for rate‐adaptive pacing arises primarily in patients with sinus node dysfunction. In the 1980s, a variety of sensors were tested as “physiological” control mechanisms, but none fulfilled the requirements of a true closed‐loop system. Attempts were made to link pacing rate to exercise intensity using parameters such as blood pH, blood temperature, respiration, the stimulus–T interval, oxygen saturation, stroke volume, and contractility. However, none of these provided sufficient feedback to reliably prevent overstimulation.

Werner Irnich built on the observation that the AV conduction time shortens under exercise as heart rate increases [9]. In three patients with atrial pacemakers, he investigated whether, at a constant atrial pacing rate, changes in AV delay correlated with exercise load, and what effect a change in pacing rate had at constant load. Within just 5–10 s of exercise, AV conduction time shortened and then remained stable across a broad pacing range. When a critical pacing rate was exceeded (overstimulation), the AV conduction time abruptly lengthened by 5%–8% (Figure 7). This provided the basis for negative feedback to the pacing rate. Irnich linked this concept to the hope that the frequency response could thus be transformed into genuine rate adaptation.

**FIGURE 7:**
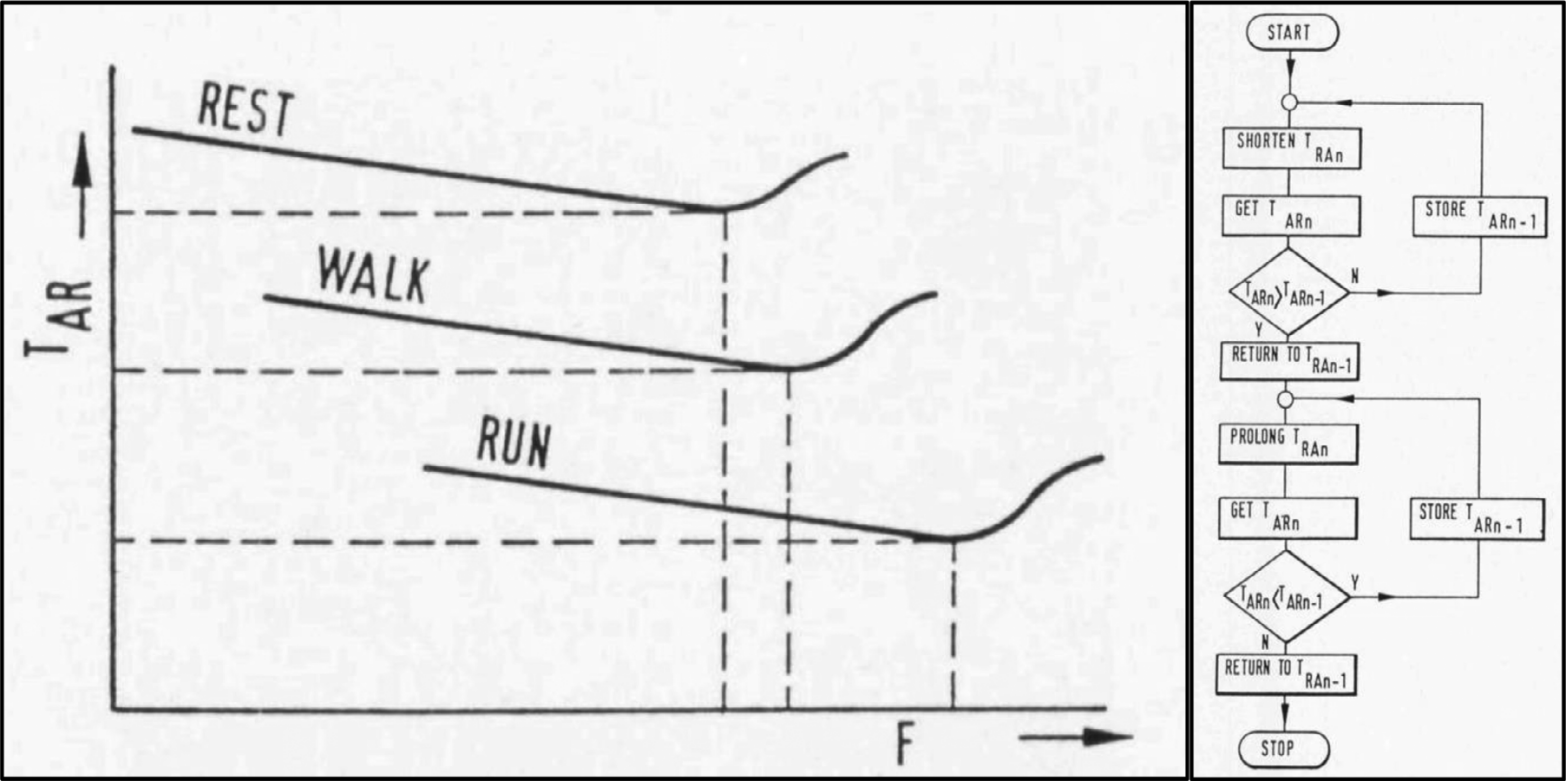
Left: Relationship between AV conduction time (TAR) and pacing rate (F). The higher the exercise level, the higher the rate at which “overstimulation” occurs. Right: Flow chart for determining the pacing rate below the threshold of “overstimulation.” Irnich W, Conrady J. A new principle of rate adaptive pacing in patients with sick sinus syndrome. Pacing Clin Electrophysiol 1988; 11:1823–1828.

The principle was patented in 1988 [10] and subsequently published in the journal PACE. Ten years later, the working group of Prof. Werner at the Department of Biomedical Engineering, Ruhr University Bochum, revisited the concept and developed it into a closed‐loop system [11]. In 1999, Herxamer filed a patent based on this approach [12]. However, rate‐adaptive systems of this kind were never implemented in an implantable pacemaker.

## 4 Curriculum Vitae of Werner Irnich

Werner Irnich was born in 1934 in Anrath, district of Kempen‐Krefeld, Gemany, together with his twin sister, as the youngest of five children of Josef and Juliane Irnich (née Mundt). His father was a secondary school teacher at the Gymnasium (grammar school) in Uerdingen, while his mother, who had also studied education, was a homemaker and mother. Werner attended the Gymnasium in Uerdingen am Rhein, where he completed his Abitur (secondary school diploma) in 1955.

After studying electrical engineering at RWTH Aachen (Dipl.‐Ing., 1960), he worked until 1968 as an assistant at the Institute of Theoretical Electrical Engineering under Prof. Dr. rer. nat. Walter Engl. In 1968, he earned his doctorate in engineering (Dr.‐Ing.) at RWTH Aachen in the field of medical technology. His dissertation was titled *“Measurements for Noninvasive Blood Pressure Monitoring”*.

From August 1968 onward, he worked under Prof. Dr. med. Sven Effert in the Department of Internal Medicine I, specializing in cardiology, at the University Hospital of RWTH Aachen—initially as an assistant, later as an senior resident scientist. He became head of the Bioelectronics Laboratory and was responsible for the care of pacemaker patients (Figure 8).

**FIGURE 8:**
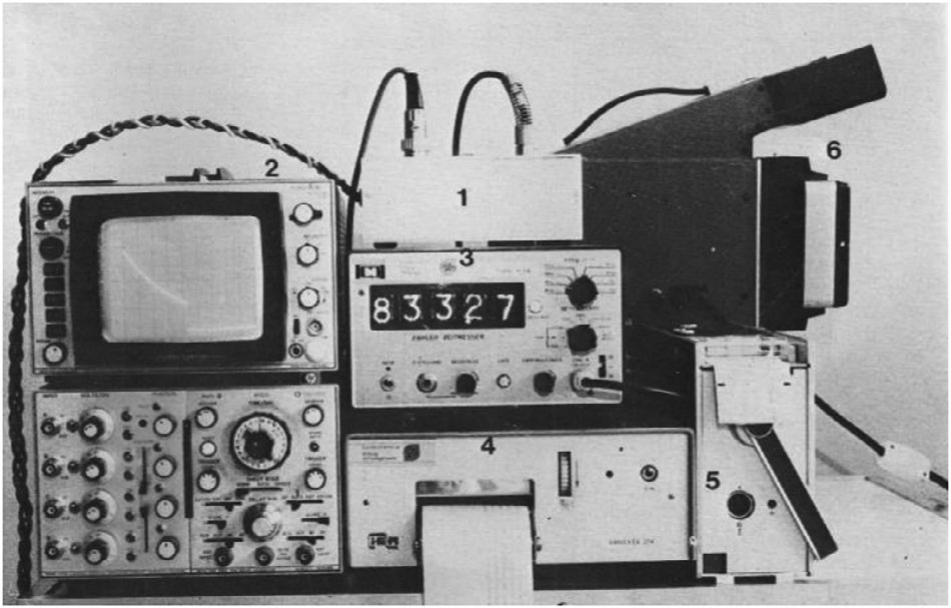
Test station for pacemaker evaluation with oscilloscope display of the stimulation impulse and interval counter. Irnich W. 50th anniversary celebration of the Medical Faculty RWTH Aachen 2016 “When medical technology came to Aachen”.

In 1975, he completed his Habilitation (postdoctoral qualification)—the first engineer to do so at the Medical Faculty of RWTH Aachen—in the field of medical measurement technology and bioelectronics, and was appointed Assistant Professor (Privatdozent). The topic of his habilitation thesis was *“Electrotherapy of the Heart”*.

This was followed by professional appointments in Aachen and Berlin and, in 1977, a research stay at Massachusetts General Hospital in the United States. In 1979, he was appointed Full Professor and became the Chair of Medical Engineering at Justus Liebig University in Giessen, where he served as director of the Institute of Medical Engineering.

His teaching and research focused on:

- Electrical stimulation in the fields of cardiac pacing and defibrillation.
- Electrical safety, with particular attention to pacemaker patients.
- Management of efficient biomedical engineering services in hospitals.
- Cardiac support systems (i.a. intra‐aortic balloon pump).
- Challenges in high‐frequency surgery.

Between 1982 and 1999, he ran the German Pacemaker Registry. In 1999, he retired. The valedictory addresses were delivered by Seymour Furman on behalf of NASPE (North American Society of Pacing and Electrophysiology) and by the author on behalf of the German Society of Cardiology (DGK). In his obituary for Seymour Furman (who died on February 20, 2006, after serving as editor‐in‐chief of PACE for more than 25 years), he recalled their first meeting at the eighth International Conference on Medical and Biological Engineering in Chicago in 1968 [13].

Werner Irnich was a member of numerous scientific societies, including the Working Group on Pacemakers of the German Society of Cardiology (DGK). From 1978 onward, he served as a member and chair of the subcommittee “Active Implants” of the German Commission for Electrical Engineering (DKE), and was a guest member of the subcommittee “Safety in Electromagnetic Fields.” He was also a member of the German Radiation Protection Commission, Subcommittee on “Non‐Ionizing Radiation,” and an elected Fellow of the IEEE (Institute of Electrical and Electronics Engineers, USA).

From 1969 to 1972, he was secretary of the German Society for Biomedical Engineering within the Association of German Electrical Engineers (VDE). Between 1972 and 1979, he served as managing editor of the journal *Biomedizinische Technik*. From 1978 to 2013, he held the position of Senior Editor of the journal *Pacing and Clinical Electrophysiology* (PACE). During this time, he published more than 70 scientific papers in PACE.

Werner Irnich was the author or co‐author of 376 journal and conference publications, and five monographs. He held 15 patents in the field of medical technology. He passed away on December 2, 2023, at the age of 89, leaving behind his wife Hanni, five children, and twelve grandchildren.

## 5 The Fundamentals of Stimulation

To illustrate the way of thinking of the engineer Werner Irnich, one of his early works from 1969 may be cited. The problem in the early years of electrical stimulation was to determine the optimal electrode size with the lowest excitation threshold voltage. *“By means of a physical abstraction, an attempt is made to explain the phenomena occurring in the determination of the excitation threshold and to draw conclusions as to how electrode size influences the threshold”* [14].

The mathematical derivation leads to the conclusion that the field strength of an impulse is the effective agent at the excitable cell membrane, and that this field strength increases the smaller the electrode radius and the thinner the non‐excitable scar tissue that surrounds the electrode as a result of a foreign body reaction. This insight, which forms the theoretical basis for the physical design and pharmacological surface coating of modern electrodes, finds perfect correspondence through in vivo measurements.

Another significant aspect of electrical stimulation is the strength–duration curve, also referred to as chronaxie–rheobase, which dates back to the work of Lapicque in 1909. Irnich emphasized its importance in a fundamental article in 1980 [15] and repeatedly highlighted its practical usefulness in publications and lectures. The rheobase denotes the minimal pulse amplitude required to elicit a response at infinite pulse duration. The chronaxie is the value at which a response is achieved at minimal pulse duration with a pulse amplitude equal to twice the rheobase (Figure 9). This point represents the condition of the lowest effective energy output. It depends on the electrode configuration and, for modern electrodes, lies closer to 0.2 ms rather than the factory default of 0.4 ms.

**FIGURE 9:**
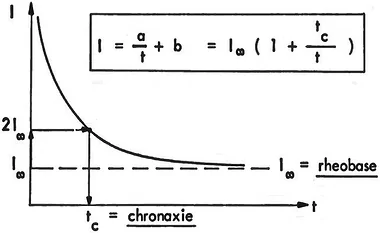
Strength–duration curve (see text for explanation). Irnich W. The chronaxie time and its practical importance. Pacing Clin Electrophysiol 1980; 3:292–301.

Irnich deeply regretted that this insight has found little application in pacemaker therapy. In an investigation of 833 explanted pacemakers from deceased patients, he found that in 33.5% of ventricular and 50.3% of atrial pacemakers the pulse duration had not been changed from the factory setting [16].

## 6 The Fundamental Law of Electrical Stimulation

In a foundational study from 1974, Irnich determined the fibrillation threshold for alternating voltage and alternating current [17]. In animal experiments, the stimulation threshold was measured using sinusoidal half‐waves, full waves, and double waves. From these results, he concluded that the stimulation effect is essentially produced by the first sinusoidal wave. Outside of the refractory period, the first wave can then re‐excite the myocardium, which can give rise to fibrillation. The stimulation threshold is approximately 35%–40% of that required for a rectangular pulse of 1 ms duration. Application of continuous sinusoidal waves above the stimulation threshold induces ventricular fibrillation. With this, Irnich laid the foundation for safety evaluations of medical devices. In a fundamental article published in 1989, he summarized his findings once again: *“An electric current through the body becomes dangerous when it generates an electric field strength of 34 V/m or more within the heart”* [18].

In 1990, he extended the fundamental law of electrical stimulation to the defibrillation of the heart. According to Irnich, stimulation and defibrillation are not fundamentally different in principle. Electrical stimulation means the action of an exogenous electric field on the membrane's permeability barrier, allowing sodium influx to occur. In contrast to stimulation, which triggers a focal excitation, defibrillation requires a homogeneous excitation of the entire myocardium to prevent the reinduction of ventricular fibrillation. The fundamental technical principle of defibrillation is to generate an electric field of 400 V/m or more within the ventricles. Moreover, a chronaxie–rheobase relationship also applies to defibrillation [19]. However, as he lamented, physicians today typically demand only generators with high stored energy (40 J or even 47 J), while generally ignoring other parameters. In Irnich's view it is often not recognized that the size of the output capacitance and the pulse duration play an important role in defibrillation efficiency [20].

## 7 The Problem of Interference Detection

*“An early contribution of Irnich was the introduction of back‐to‐back Zener diodes at the pacemaker input to reduce low‐frequency demodulation products.”* These Z‐diodes were applied for voltage limiting, overload protection (e.g., during defibrillation), and voltage stabilization. *“Internationally, Irnich was a pioneer in developing protection against electromagnetic interference. All international rules and standards for shielding pacemakers and defibrillators from electromagnetic fields are based on Irnich's work. Whenever the electromagnetic protection of implants was discussed—also internationally—Irnich was the leading authority”* [21]. As early as 1976, Irnich's colleague de Bakker had developed a patent for an interference detection circuit for synchronizable pacemakers [22]. In the first issue of the journal “*Pacing and Electrophysiology*” in 1978, he published a groundbreaking article on the topic of “*state of the art in interference detection*” [23]. With the method he proposed, the problem could be overcome through simple time‐domain analysis. In the following years numerous papers on electromagnetic interference were published, including those on mobile phones [24], surveillance systems [25] and magnetic resonance imaging [26]. Irnich himself filed the patent *“synchronizable pacemaker with fault detection circuit”* with the European Patent Office in 1984. *“The interference detection circuit according to the invention is particularly effective because, unlike known detection circuits, it takes into account the typical structure of an intracardiac signal, regardless of whether it is derived atrially or ventricularly”* [27].

This approach gave priority to waveform analysis over detection thresholds of filtered signals. Irnich reaffirmed this view in his contribution to the special issue of *Herzschrittmacher und Elektrophysiologie* [28], published on the occasion of his 70th birthday: *“Cardiac signals, unlike 50 Hz signals, are too heavily attenuated. There is no pacemaker or ICD that can morphologically distinguish between useful and interference signals”* [29]. As an example, he cited the ventricular electrograms of two pacemakers, in which the useful signal was reduced to 80% and even as low as 31% due to band‐pass filtering (Figure 10).

**FIGURE 10:**
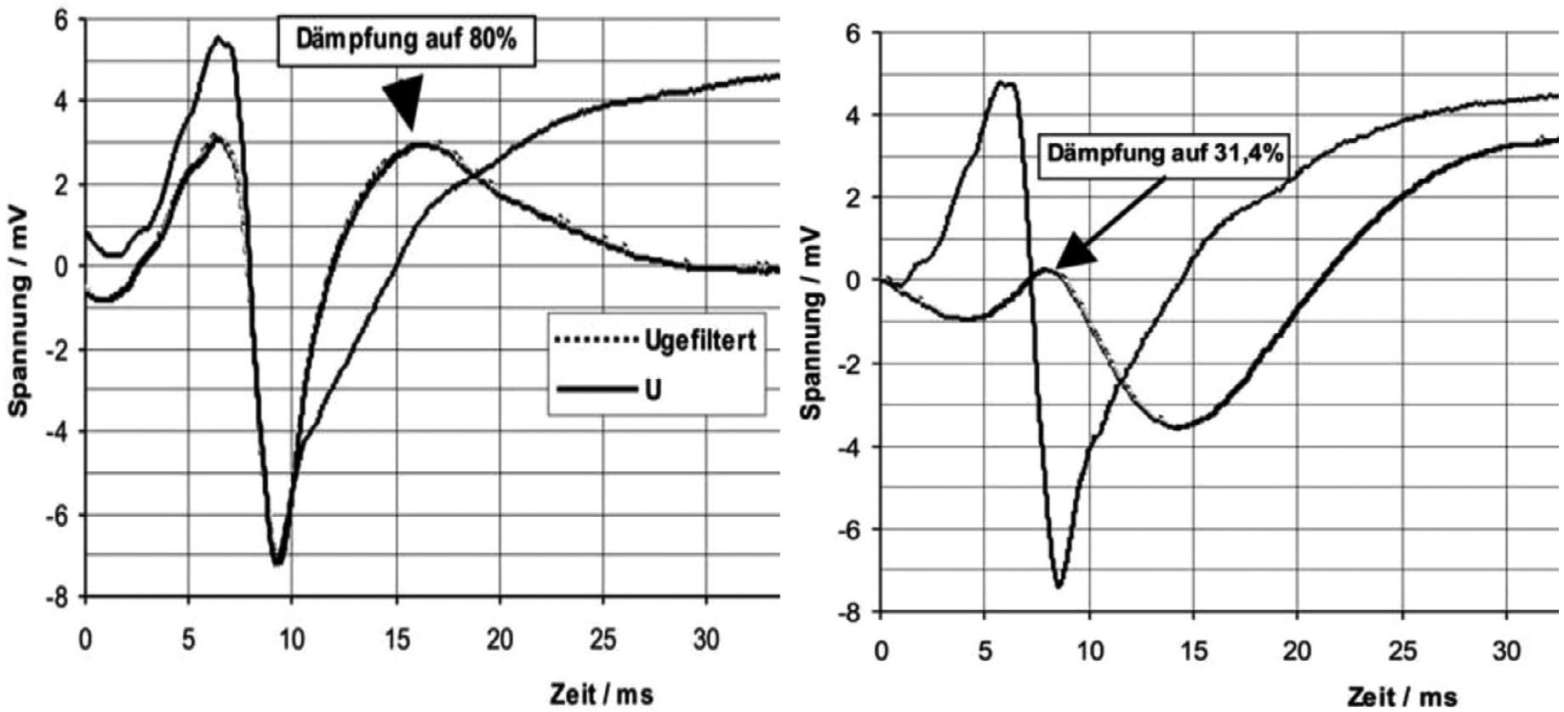
Unipolar ventricular electrogram without and with band‐pass filtering. Irnich W. Störbeeinflussung von implantierbaren Herzschrittmachern und Defibrillatoren. Herzschr Elektrophys 2004; 15:9–21.

## 8 The Central Pacemaker Registry

In 1982, Werner Irnich established the German Central Pacemaker Registry (“Deutsches Zentralregister Herzschrittmacher”), which he directed until 1999. The registry systematically collected and analyzed clinical and technical data on implanted pacemakers, replacements, and explantations throughout Germany. The registry was based on the European Pacemaker Identification Card, which provided a standardized documentation system across implanting centers. Participation was voluntary, but despite this limitation the registry consistently captured approximately 30%–40% of all procedures performed in Germany. Although incomplete, the registry thus represented a reliable cross‐section of real‐world clinical practice in Germany and, by virtue of its size and duration, became the largest database of its kind worldwide. It offered valuable insights into device use, complication rates, and long‐term performance, and served as a benchmark for both clinical outcomes and technical development.

In 1974, Irnich and Effert observed that dual‐chamber pacing was realized in only about 1% of patients, although under optimal conditions it should have been feasible in up to 70% [30]. By the time Irnich retired in 1999, the proportion had risen to 52% (Figure 11, left) [31], and by 2022 it had reached 75%, with an additional 8% accounted for by CRT‐P systems [32]. Thus, more than fifty years later, the vision formulated in 1974 can be considered fulfilled. At the same time, the non‐lead related complication rates—which in 1985 were still close to 25%—declined to less than 5% by the time of Irnich's retirement (Figure 11, right) and are currently estimated to be as low as 1% per year. This dramatic improvement highlights the central role of quality assurance in advancing patient safety and therapeutic outcomes.

**FIGURE 11:**
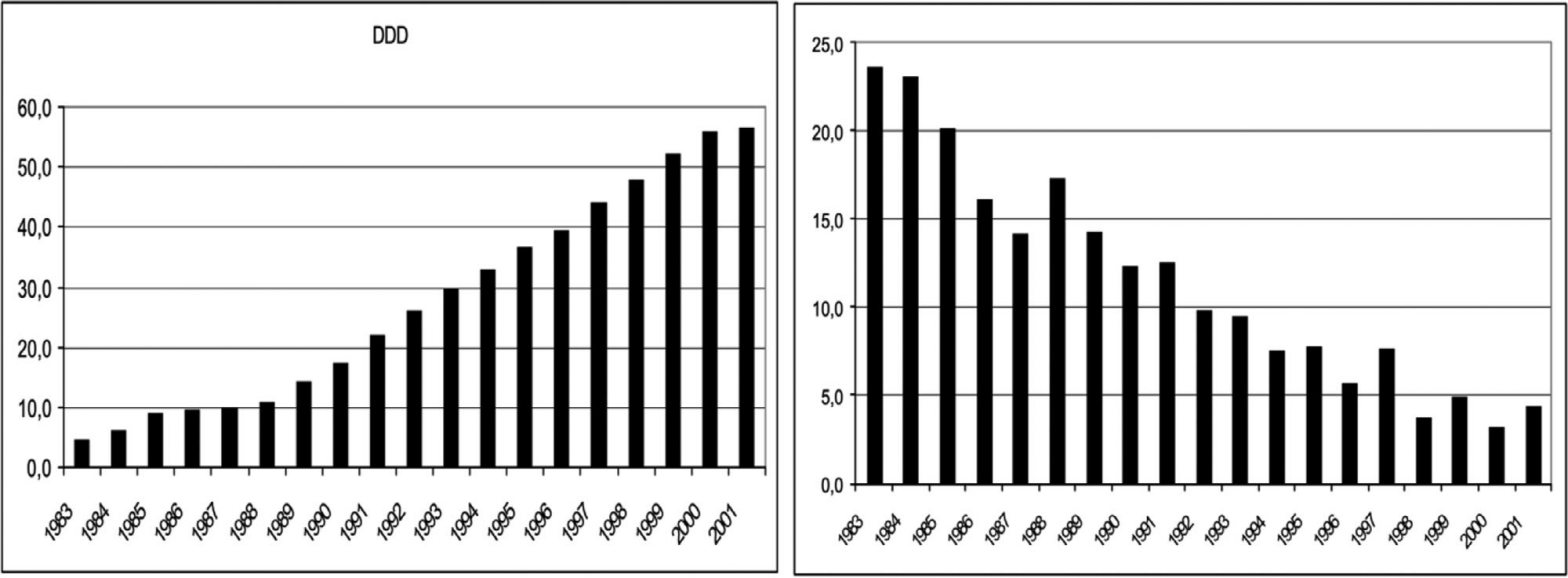
Left: Proportion of DDD pacemakers; Right: Frequency of complications (inhibition by muscle potentials, extracardiac stimulation, penetration, erosion, infection, wound pain). Deutsches Zentralregister Herzschrittmacher, Tabellen und Graphiken 1982–2001. https://pacemaker‐register.de/wp‐content/uploads/tabellen_graphiken_82‐01.pdf.

## 9 Laudation and Obituary

On the occasion of Werner Irnich's 70th birthday, *Herzschrittmacher und Elektrophysiologie* published the following laudation by Prof. Gerd Fröhlig and Dr. Wilhelm Fischer [33]:

*“Whoever wishes to learn how pacing and sensing truly function can do so from his famous reviews; the most extensive body of data on electromagnetic interference safety in electronic implants originates from his laboratory; in his didactic fundamental articles, he argued for a deeper understanding of physiological and technical interrelations; as an engineer, he was an advocate for pacemaker patients.”*

*“It is no wonder that he was not always considered convenient—sometimes even inquisitorial. His guiding principle, that only what is precisely defined and correctly named can be properly understood, often collided with what clinicians dismissed as a ‘lack of clinical relevance’—an attitude that frequently reflected their reluctance to engage in profound analysis.”*

Little more needs to be added.

Werner Irnich embodied the close collaboration between engineers and physicians in the field of cardiac electrotherapy. His theoretical work on chronaxie–rheobase relationships and on electrode surface properties, confirmed by experimental data, continues to provide the foundation of modern cardiac stimulation.

His concept of the “optimal pacemaker” was visionary and shaped an entire era; it remains relevant today, with the added refinement of optimal stimulation site selection.

The establishment of the German Pacemaker Registry was a milestone in quality assurance. Unlike today's systems, its purpose extended beyond documentation to the early detection of systemic failures and device deficiencies.

We will always honor Professor Werner Irnich with gratitude and recognition for his lasting contributions to the field of cardiac pacing and stimulation.

## Funding

The authors have nothing to report.

## Consent

No informed consent from patients is required for this historical review.

## Conflicts of Interest

The authors declare no conflicts of interest.
